# Perspectives on the Treatment of Lumbar Disc Degeneration: The Value Proposition for a Cell-Based Therapy, Immunomodulatory Properties of Discogenic Cells and the Associated Clinical Evaluation Strategy

**DOI:** 10.3389/fsurg.2020.554382

**Published:** 2020-12-16

**Authors:** Lara Ionescu Silverman, Will Heaton, Niloofar Farhang, Lindsey Hart Saxon, Galina Dulatova, Daniel Rodriguez-Granrose, Flagg Flanagan, Kevin T. Foley

**Affiliations:** ^1^DiscGenics Inc., Salt Lake City, UT, United States; ^2^Department of Neurosurgery, University of Tennessee Health Science Center, Memphis, TN, United States; ^3^Semmes-Murphey Clinic, Memphis, TN, United States

**Keywords:** opioids, disc degeneration, regenerative medicine, cell therapy, low back pain, IDCT

## Abstract

Low back pain (LBP) is a serious medical condition that affects a large percentage of the population worldwide. One cause of LBP is disc degeneration (DD), which is characterized by progressive breakdown of the disc and an inflamed disc environment. Current treatment options for patients with symptomatic DD are limited and are often unsuccessful, so many patients turn to prescription opioids for pain management in a time when opioid usage, addiction, and drug-related deaths are at an all-time high. In this paper, we discuss the etiology of lumbar DD and currently available treatments, as well as the potential for cell therapy to offer a biologic, non-opioid alternative to patients suffering from the condition. Finally, we present an overview of an investigational cell therapy called IDCT (Injectable Discogenic Cell Therapy), which is currently under evaluation in multiple double-blind clinical trials overseen by major regulatory agencies. The active ingredient in IDCT is a novel allogeneic cell population known as Discogenic Cells. These cells, which are derived from intervertebral disc tissue, have been shown to possess both regenerative and immunomodulatory properties. Cell therapies have unique properties that may ultimately lead to decreased pain and improved function, as well as curb the numbers of patients pursuing opioids. Their efficacy is best assessed in rigorous double-blinded and placebo-controlled clinical studies.

## Low Back Pain and Disc Degeneration

Low back pain (LBP) is a leading cause of disability worldwide (1), and the leading cause of years lived with disability in developed countries (2). The 2010 Global Burden of Disease Study estimated that LBP is among the top 10 diseases and injuries that account for the highest number of DALYs (disability-adjusted life years) worldwide (3). In the US, low back pain (LBP) affects 12–30% of US adults at a given time (4) and the annual expenditure to treat LBP is estimated to be over $100 billion (1), creating a significant burden on the economy as well as individual patients. Not only is there direct expense from treating LBP, but also one third of all occupational musculoskeletal injuries and illnesses resulting in work disability are attributed to LBP (5). This means that there is a significant loss of productivity and indirect costs from missing work. Current treatment options for patients with LBP may prove unsuccessful in alleviating pain or improving disability, forcing patients to look for other ways to seek relief. Often, this means turning to prescription opioids for pain management in a time when opioid usage, addiction and drug-related deaths are at an all-time high (https://www.cdc.gov/drugoverdose/epidemic/index.html). In fact, LBP is the most common, non-cancer reason for opioid prescription in the United States (6, 7). According to the CDC, there were 168 million prescriptions of opioids in 2018 (8), and nearly 68,000 opioid overdose deaths in the U.S. that year (9). Driving down the use (and abuse) of opioids by developing safer, more effective treatments for LBP is a critical task for the healthcare community.

A major cause (up to 39%) of LBP is disc degeneration (DD) (10–12), a condition in which the intervertebral disc breaks down and causes pain. The intervertebral disc, which is comprised of a gelatinous central nucleus pulposus and an outer annulus fibrosus, is avascular, hypoxic, and hypocellular (13), making it perhaps more prone to degenerative conditions. The condition features an imbalance in cytokines that leads to tissue breakdown and direct pain sensation (14). This breakdown is exacerbated by the depleted capacity of local cells to produce new extracellular matrix molecules as well as an imbalance in anabolic/catabolic signals such as matrix metalloproteinases in the tissue (13). These changes result in global tissue structure damage that may be manifested by acute and chronic pain, and may eventually result in structural failure, requiring surgical intervention. Also, degenerated discs are characterized by an upregulation of inflammatory cytokines such as interleukins and tumor necrosis factor-alpha (14). Other properties of DD include pathologic innervation, vascularization, and changes to the endplate.

Many risk factors are associated with developing lumbar DD, including genetic predisposition, acute injury, and modifications to adjacent levels (such as fusion surgeries) that result in abnormal biomechanics (15). Clinically, patients who experience chronic LBP seek medical attention to reduce their pain and disability as well as to increase their quality of life. Treating low back pain is a complex medical task, further challenged by the interplay of psychosocial factors, chronicity, and comorbidities (16). MRI is often used to evaluate the lumbar spine for Modic changes and Pfirrmann scores of lumbar discs (17), which indicate DD; however such imaging does not always correlate with patient symptomatology and therefore is not the sole driver of therapy selection (18). Clinical presentation must correlate with the radiographic findings. Discography may also help diagnose symptomatic lumbar disc degeneration, but has been used less in recent years due to concerns around worsening the disease from the procedure itself (19).

Currently, treatment of LBP that reduces the need for surgical intervention and opioid prescription is an unmet medical need that is recognized by the medical community (20, 21). Historically, epidural steroid injections were utilized as a non-surgical method to treat DD; however, lack of definitive efficacy in controlled studies has decreased the use of this treatment when patients lack radiculopathy (22). Approaches such as nerve stimulation and nerve ablation have been considered but are not yet well-proven for the treatment of DD. In 2018, a Global Spine Care Initiative outlined that treatments for LBP should be limited to non-interventional treatments (yoga, massage, and pain medications) (23), as other approaches do not have sufficient evidence of efficacy. Currently, there are no non-invasive treatments available that have shown robust clinical evidence in reducing pain and disability and increasing quality of life. Further, no treatments exist that have proven the ability to eliminate the need for surgical interventions. One common surgical intervention is fusion surgery, which has mixed outcomes and can lead to long-term opioid abuse and addiction, especially in patients who sought pain relief from opioids prior to surgery (24, 25). Patients suffering from DD need a proven, non-invasive alternative for treating LBP.

## Cell Therapy to Treat LBP

LBP induced by DD has been historically challenging to treat. Small molecules and biomaterials have been clinically evaluated but have shown mixed success. For example, growth factor GDF-5 did not show success in double-blinded clinical trials (26), and various anti-inflammatory proteins including tumor necrosis factor and IL-6 inhibitors have shown mixed success even when patients and providers were blinded in clinical trials (27). The anti-inflammatory proteins have limited evidence of having long-term effects, possibly due to their short half-lives ranging from ~3 to 20 days dependent on the molecule (28–31), which is much less than what is considered chronic LBP (>12 weeks) (32). Similarly, biomaterials have not shown robust success. While some pain reduction has been seen, the outcomes are mechanical in nature and lack any biologically active component that could cause regeneration (33, 34). Additionally, complications are prevalent with biomaterials, including biomaterial leaking out of the disc and causing additional pain, and excess stiffness causing endplate fracture (35, 36). As a result, neither anti-inflammatory proteins nor biomaterials present an ideal approach to treating DD.

More recently, delivery of a live cell population into the disc is under consideration as an option that can fill this treatment need (37). There are several reasons a cell therapy may be more successful at treating LBP than the other approaches. First, cells may have a longer residence time than small molecules, some of which have half-lives of <24 h (38–40). Also, cells can have multiple mechanisms of action that can more appropriately tackle a complex disease such as DD. Finally, cells can respond to the local micro-environment, suitably replacing the local cell population and potentially promoting a more normal local milieu through their paracrine signaling. However, cell therapies also face some specific challenges in the disc, particularly due to the harsh environment (low pH and oxygen) that may inhibit proper cellular functionality (41).

Cell therapy-based treatments also face notable challenges associated with commercializing such medicines. Attaining manufacturing consistency and suitable scale is a challenge with live cells, and depending on the FDA pathway being utilized, regulators may expect drug-style compliance to the Code of Federal Regulations (CFR). The FDA regulates cell therapies under one of two pathways, which delineates their level of involvement. Therapies that are minimally manipulated, homologous or meet other narrow criteria have limited regulatory oversite and fall within Section 361 of FDA's 21 CFR Part 1271 regulation. Therapies that contain cells that have been substantially changed, or manipulated through the manufacturing process, are regulated under Section 351 of 21 CFR 1271, and require more regulatory involvement (42). The section 351 regulatory path closely follows that of traditional pharmaceutical products, including preclinical studies, manufacturing/quality oversight, and clinical trial execution (42). The section 351 route also involves designating a specific mechanism of action, which is often difficult to achieve as large rates of non-responding patients in cell therapy clinical trials make the mechanism of action difficult to define (43).

Nonetheless, despite these potential environmental, manufacturing and regulatory hurdles, multiple cell therapies (43), including an allogeneic cell therapy treatment our group is developing called IDCT, are in clinical evaluation. Such treatments hold promise as a potential therapeutic approach to curb opioid abuse among patients suffering from the condition.

## Overview of Injectable Disc Cell Therapy (IDCT) and Immunomodulatory Evaluation

IDCT is novel cell therapy under clinical evaluation for the treatment of LBP caused by DD. The active ingredient is a live population of Discogenic Cells, which originate from donated adult intervertebral disc tissue. After undergoing a proprietary growth process, the cells are frozen for storage and thawed immediately prior to use. The cells are delivered directly into the painful, degenerated disc through a needle placed via fluoroscopic guidance. Discogenic Cells are allogeneic in nature as the starting material is from a donor and the cells are expanded, modified, and subsequently frozen, and then delivered to different people (in contrast to autologous therapies, which begin and end with the same patient). Also, because the donor cells in IDCT originate from the disc and are then reintroduced into the recipient's disc, the product is homologous. Due to the fact that the cells are more-than-minimally manipulated, the manufacture of these cells requires a high level of regulatory oversight to ensure proper standards are being met (21 CFR 210/211). During this process, large quantities of Discogenic Cells can be produced in a single lot, making the treatment scalable to the DD patient population.

During the growth process, cells exhibit phenotypic changes from that of cells found in the native disc tissue. Specifically, the cells lose expression of CD24 (44), which is a marker for nucleus pulposus cells (45). Also, Discogenic Cells have a unique surface marker expression profile that includes high expression of CD73, CD90, and HLA-ABC and a low expression of CD34 and HLA-DR/DQ/DP (44). The cells generate the extracellular matrix found within native intervertebral disc tissue, including proteoglycan and collagen. The matrix production has been measured *in vitro* using techniques such as histology (44), PCR and biochemical assays. Further, evaluation of the cells in animal models of disc degeneration demonstrated normalization of disc height and tissue architecture in both rabbits (44) and dogs (46).

We hypothesize that improvement in disc height *in vivo* may alleviate compression on nerves which cause pain and may modify the local microenvironment in a way that may reduce overall catabolic changes, and therefore inflammation and pain. Improved disc height observed in animal models when using other cell therapies has shown correlation to a reduction in pain when tested in human clinical trials, although the results lack robustness (47–49).

Another important aspect of treating disc degeneration is to address the inflammation within the disc that may contribute directly to pain sensation and tissue breakdown. Because the treatment is allogeneic, understanding the potential immunogenicity of the treatment is critical prior to clinical evaluation. Confirming the absence of surface markers CD40, CD80, and CD86, which are required for effector T cell induction (50), is important in a potential allogeneic cell therapy. Also, activated T-cell assays can evaluate the potential for immune rejection and also assess whether the therapy has immunomodulatory effects. In these studies, we evaluated these key properties prior to clinical evaluation.

### Methods

Intervertebral disc tissue was obtained from consented male and female donors through DonorConnect (Murray, Utah) and cells were harvested and processed into Discogenic Cells as described in (44). In order to mitigate the risks of adventitious agents being present in the tissue, a Medical Director reviewed donor medical records to determine donor eligibility according to government guidelines, which includes a review of serology and risk factors. Ten lots of Discogenic Cells were analyzed for cell surface antigen expression by flow cytometry using the following fluorescence-conjugated mouse antihuman monoclonal antibodies: CD40, CD80, CD86 (BD Biosciences, San Jose, CA). Appropriate isotype controls were run in parallel. Also, cell lines known to be positive for each antigen were procured from ATCC (Manassas, Virginia) and evaluated to ensure that a positive signal was attainable. The cells were blocked in PBS containing 0.5% human serum albumin (Baxter, Deerfield, IL) and 25 μg/mL Fc-block (BD Biosciences) for 10–20 min. Cells were subsequently stained with antibodies for 30–60 min at 4°C protected from light and subsequently washed and resuspended in PBS containing 0.5% human serum albumin. 7-AAD (BD Biosciences) was used as a dead cell exclusion marker. A minimum of 10,000 events were collected on a Cytoflex Flow Cytometer (Beckman Coulter, Indianapolis, IN, USA) using CytExpert Software for data acquisition and FlowJo Software for analysis.

Immunomodulatory properties of IDCT were analyzed by testing the ability of IDCT to inhibit proliferation of T-cells from two different donors. Healthy peripheral blood mononuclear cells were obtained from two vendors (Precision for Medicine and STEMCELL Technologies) and T-cells were isolated from them using the EasySep^TM^ Human CD4+ T Cell Isolation Kit (STEMCELL Technologies, Vancouver, BC) according to manufacturer's instructions. Subsequently, T-Cells were activated using CD3/CD28 T-cell activator (STEMCELL Technologies) and cultured in T-cell expansion media (Immunocult XF T Cell Expansion Medium, STEMCELL Technologies) for 6 days according to manufacturer's instructions. During this culture time, Discogenic Cells were plated at a density of 100,000 cells/well in recovery media (DMEM/F-12 with 15% FBS, 50 μg/mL gentamicin, and 2.5 μg/mL Amphotericin B) in a 96-well plate and allowed to attach and equilibrate for 3 days. Discogenic Cells were then mitomycin treated (40 μg/mL) for 2 h at 37°C and 5% CO_2_. T-cells which had been cultured and expanded for 6-days were added to mitomycin treated Discogenic Cells at 100,000 cells/well and co-cultured for 3 days. On the third day of co-culture, BrdU was added to a concentration of 10 μM, and cells were incubated at 37°C, 5% CO_2_ for 3 h prior to analysis of proliferation of T-cells using a colorimetric BrdU assay (MilliporeSigma, Burlington, MA), according to manufacturer's instructions. A students *T*-test was used to determine differences between groups with significance set to *p* < 0.05.

### Results and Discussion

**Figure 1:**
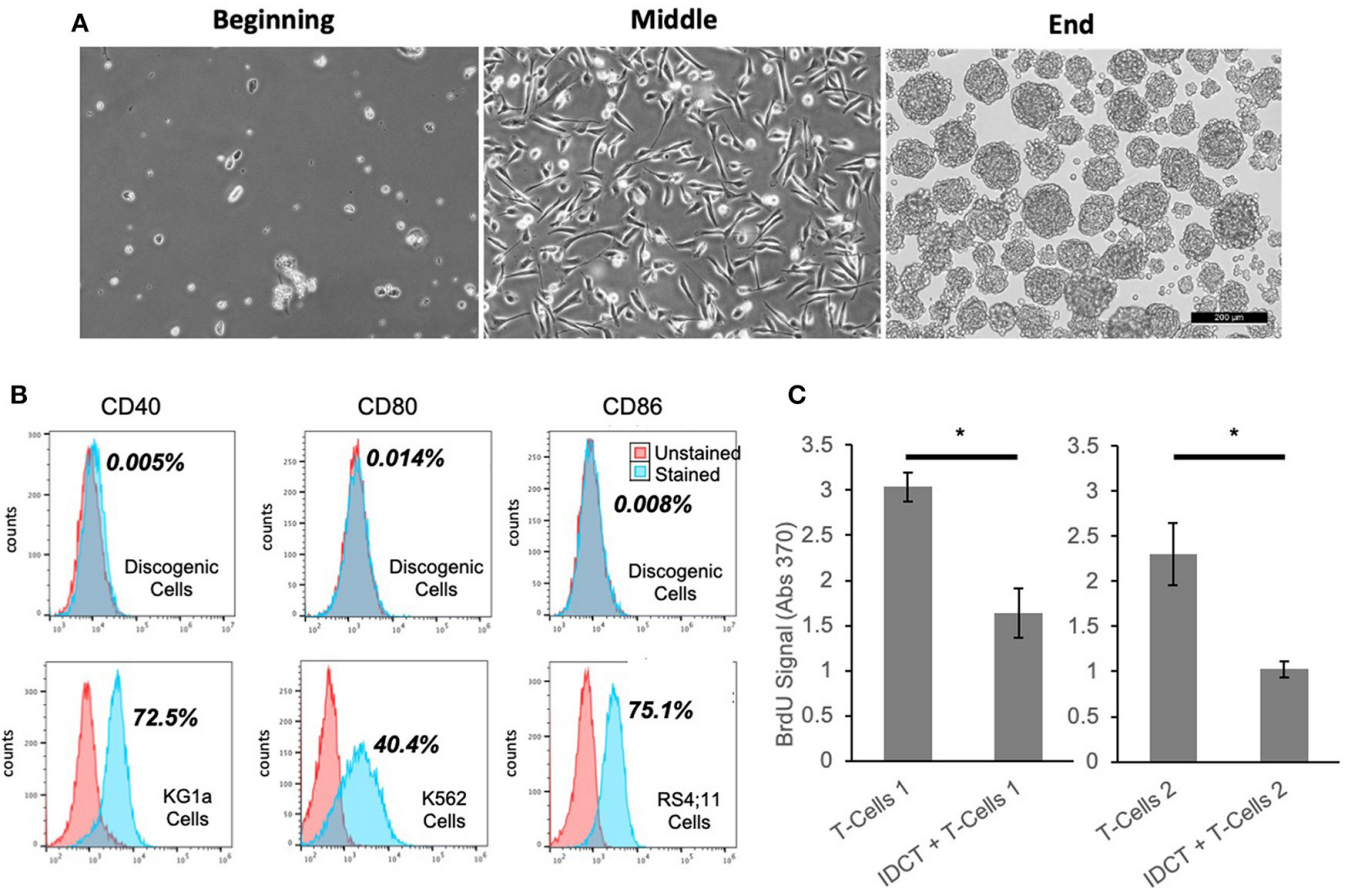
Unique properties of discogenic cells. **(A)** The morphology of the cells change from the beginning to the end of the process, where the Discogenic Cell phenotype is attained (Scale bar is 200 μM; one representative set of images shown from *n* = 10). **(B)** Flow cytometry of Discogenic Cells shows a lack of co-stimulatory markers CD40, CD80, and CD86, which if expressed could induce T-cell activation and rejection. Ten Discogenic Cell samples tested; one representative dataset shown. Control cells show positive expression of the markers, validating the method. The quantitative result for the percentage of stained cells positive for each marker is shown in bold next to the histograms. **(C)** Measurement of proliferation of CD3/CD28 stimulated T-Cells from two different donors, with or without Discogenic Cell co-culture. Proliferation was measured by BrdU assay. Overall this indicates the ability of IDCT to inhibit T-cell proliferation thus indicating it has potential immunomodulatory capabilities (*n* = 5 technical replicates, *p < 0.05 by Student's *T*-Test).

Discogenic Cells were generated and achieve normal morphology (Figure 1A). Flow cytometry revealed lack of expression of co-stimulatory markers CD40, CD80, and CD86 (50) (Figure 1B). This absence of T cell induction is hypothesized to lead to minimal immunogenicity of Discogenic Cells. Next, when combined with activated human T-cells, Discogenic Cells did not increase T-cell expansion, but in fact suppressed T-cell expansion (Figure 1C), demonstrating a lack of immunogenicity in this assay as well as an immunomodulatory effect on T-cells. Such findings may indicate that Discogenic Cells could directly modulate pain sensation in a degenerated disc environment.

IDCT cells are derived from the tissue type they are intended to treat, which may result in better outcomes than using cells not accustomed to the disc environment. These Discogenic Cells, which differ from the cells originally obtained from the disc tissue, are both immunomodulatory and regenerative and thus have the potential to impact the pathophysiology of disc degeneration.

## Next Steps: Ongoing Clinical Evaluation of IDCT

Treatment of patients with lumbar disc degeneration utilizing cell therapy has been explored in prior clinical studies, showing the feasibility of this approach. In early-stage human clinical trials, multiple types of mesenchymal stem cells (51–53) and chondrocytes (48, 54, 55) have been used. These studies have shown the injection delivery method to be feasible and the treatments to be safe. In small, open-label studies without controls, the treatments reduced pain and, in some instances, reduced Pfirrmann scores (52, 53). Given the strong placebo effect that can be encountered when evaluating treatments for pain, blinded studies that utilize control arms are needed to evaluate the true effects of cell therapy. For example, in a study utilizing a subpopulation of mesenchymal stromal cell (MSCs), pain was modestly reduced compared to control, but none of the regenerative parameters [such as Pfirrmann score, disc height or magnetic resonance imaging (MRI)] improved over time (56). Thus, while the approach seems promising, the ideal cell type remains elusive.

Preliminary safety and efficacy of IDCT is under evaluation in 60 patients with single-level, symptomatic DD across 14 sites (clinicaltrials.gov identifier NCT03347708). Institutional review board approval has been obtained. The subjects who meet all eligibility criteria are being randomized to one of four treatment cohorts: low dose IDCT (*n* = 20), high dose IDCT (*n* = 20), vehicle (*n* = 10), and placebo (*n* = 10). Each subject receives a single intradiscal injection of his or her assigned treatment into the target symptomatic lumbar intervertebral disc. The delivery is through a needle placed percutaneously into the disc using fluoroscopic guidance, so no surgical procedure is needed.

Following treatment, there is a 1-year period of subject observation and evaluation before evaluating the data, with a 1-year extension period to gather additional data. We are exploring a number of endpoints that would allow for a determination of whether a single injection of IDCT can safely and effectively treat symptomatic lumbar DD patients. For all assessments, patients are instructed to maintain their long-term chronic pain medication usage, and refrain from taking acute pain medications for 24 h before each assessment.

In this study, we evaluate patient-reported outcomes to assess whether a single injection of IDCT can reduce pain and disability, and improve quality of life. Pain is evaluated using the Visual Analog Score (VAS), which evaluates pain from “no pain at all” (score of 0) to “worst imaginable pain” (score of 100). This tool, used since 1923, is sensitive to treatment effects and correlates positively to other self-reporting measures of pain intensity (57). Disability is measured via the Oswestry Disability Index, which is a 10-section questionnaire that takes 3–5 min to complete, and is a commonly used outcome-measure for low back pain patients (58). Finally, the EQ-5D questionnaire is being used to measure quality of life; it has been widely used to assess low back pain patients in prior studies (59).

Because the placebo effect can affect patient-reported outcomes, we are also exploring some less subjective, behavior-based measures. First, we are assessing patients for the “Timed up and Go” (TUG) test, which measures how long it takes for a patient to stand up from a chair, walk three meters, and return (60). A faster TUG time is thought to indicate that the patient has less pain and disability (61, 62). We are also evaluating whether there is a decrease in pain medication usage after a single injection of IDCT. A reduction in pain medication might indicate that IDCT has the potential to drive down opioid use and abuse, which is a major public health problem in the US (63). Changes in pain medication usage have been noted in other studies, such as the Phase II study for lumbar DD by Mesoblast (*data not published, based on press release*).The final behavior-based assessment is to evaluate time to subsequent spine intervention (discectomy, fusion, etc.) to see if a single injection of IDCT can delay the need for additional procedures.

Additionally, we are assessing structural changes to the spine that may serve as surrogates to patient reported or behavior-based outcomes. Sequential X-ray images of the spine taken prior to treatment and over the course of 2 years will be evaluated for changes in angular and translational motion, as well as in disc height. Also, sequential MRI images (T1, T2) will be evaluated for changes to Pfirrmann score (17) and Modic score, as well as disc height and disc volume. Where possible, the exploratory MRI sequences T1-rho and T2 relaxometry will be evaluated. Upon unblinding, radiographic changes will be associated with patient-reported outcomes to better understand the mechanism for pain/disability improvement, should it be identified. Imaging may also be proposed as a surrogate endpoint for future trials.

Concurrent with the US Phase I/II study, the Pharmaceuticals and Medical Devices Agency (PMDA) has allowed DiscGenics a Clinical Trial Notification (CTN) to execute a double-blind, sham-controlled study in Japan at 6 sites (clinicaltrials.gov identifier NCT03955315). The study will enroll 38 subjects with single-level, symptomatic lumbar DD. Each subject will be randomized to receive a single injection of either low dose IDCT, high dose IDCT, or sham procedure. They will be followed for 6 months with a 6-month extension to gather additional data. The same clinical outcomes measures described above will be utilized, along with a Japan-specific pain score (JOABPEQ).

## Conclusion

Disc degeneration is a major cause of LBP and is associated with disability worldwide. Current treatment options have limited efficacy. Unfortunately, this means that patients suffering from this condition often turn to opioids to manage their pain. No treatment has been found for DD that addresses the underlying problems of tissue architecture breakdown and an imbalance of cytokines. Cell therapies, which offer the potential to help regenerate disc tissue and moderate intradiscal inflammation, present a potentially viable option for treating DD. Several cell therapy clinical studies have been completed and more are ongoing, including the evaluation of IDCT. Rigorous clinical evaluation of candidate treatments must be performed, given the bias that may occur when evaluating subjective outcomes such as self-reported pain levels. Also, improved imaging techniques and new diagnostic tools will facilitate the development of better means for assessing this disorder. Given their potential to modify the biologic processes underlying disc degeneration, cell therapies hold the promise of being an effective treatment option for DD that mitigates pain and disability. Following the approval of such treatments, patients may no longer need to use opioids to manage their LBP.

## Data Availability Statement

The datasets generated for this article are not readily available because the datasets are confidential. Requests to access the datasets should be directed to Lara Silverman, lara@discgenics.com.

## Author Contributions

LS, NF, LH, and KF contributed writing to the manuscript. WH, NF, GD, and DR-G performed laboratory studies and contributed to figures. FF and KF provided guidance and reviewed the manuscript. All authors agreed to be accountable for the content of this work and reviewed the final content prior to publication.

## Conflict of Interest

The authors declare that this study received funding from DiscGenics Inc. The funder had the following involvement with the study: study design, collection, analysis, interpretation of data, the writing of this article and the decision to submit it for publication.
